# Differential DNA Methylation Encodes Proliferation and Senescence Programs in Human Adipose-Derived Mesenchymal Stem Cells

**DOI:** 10.3389/fgene.2020.00346

**Published:** 2020-04-15

**Authors:** Mark E. Pepin, Teresa Infante, Giuditta Benincasa, Concetta Schiano, Marco Miceli, Simona Ceccarelli, Francesca Megiorni, Eleni Anastasiadou, Giovanni Della Valle, Gerardo Fatone, Mario Faenza, Ludovico Docimo, Giovanni F. Nicoletti, Cinzia Marchese, Adam R. Wende, Claudio Napoli

**Affiliations:** ^1^Department of Pathology, Division of Molecular & Cellular Pathology, University of Alabama at Birmingham, Birmingham, AL, United States; ^2^Department of Advanced Clinical and Surgical Sciences, University of Campania Luigi Vanvitelli, Naples, Italy; ^3^IRCCS SDN, Naples, Italy; ^4^Department of Experimental Medicine, Sapienza University of Rome, Rome, Italy; ^5^Department of Veterinary Medicine and Animal Productions, University of Naples Federico II, Naples, Italy; ^6^Multidisciplinary Department of Medical, Surgical and Dental Sciences, Plastic Surgery Unit, University of Campania Luigi Vanvitelli, Naples, Italy; ^7^Clinical Department of Internal Medicine and Specialistics, University of Campania Luigi Vanvitelli, Naples, Italy

**Keywords:** Whole-genome DNA methylation, stem cell biology, regenerative medicine, computational biology, 5′-azacitidine, epigenomics and epigenetics, cellular reprogramming

## Abstract

Adult adipose tissue-derived mesenchymal stem cells (ASCs) constitute a vital population of multipotent cells capable of differentiating into numerous end-organ phenotypes. However, scientific and translational endeavors to harness the regenerative potential of ASCs are currently limited by an incomplete understanding of the mechanisms that determine cell-lineage commitment and stemness. In the current study, we used reduced representation bisulfite sequencing (RRBS) analysis to identify epigenetic gene targets and cellular processes that are responsive to 5′-azacitidine (5′-AZA). We describe specific changes to DNA methylation of ASCs, uncovering pathways likely associated with the enhancement of their proliferative capacity. We identified 4,797 differentially methylated regions (FDR < 0.05) associated with 3,625 genes, of which 1,584 DMRs annotated to the promoter region. Gene set enrichment of differentially methylated promoters identified “phagocytosis,” “type 2 diabetes,” and “metabolic pathways” as disproportionately hypomethylated, whereas “adipocyte differentiation” was the most-enriched pathway among hyper-methylated gene promoters. Weighted coexpression network analysis of DMRs identified clusters associated with cellular proliferation and other developmental programs. Furthermore, the ELK4 binding site was disproportionately hyper-methylated within the promoters of genes associated with AKT signaling. Overall, this study offers numerous preliminary insights into the epigenetic landscape that influences the regenerative capacity of human ASCs.

## Introduction

Adipose-derived mesenchymal stem cells (ASCs) represent a population of self-renewing and multipotent cells that reside in the vascular stroma of adipose tissue; under specific conditions, they are capable of differentiating into cellular phenotypes that resemble adipocytes, myocytes, chondrocytes, and osteocytes (Patrikoski et al., 2019). This indigenous cell population is known to play a central role in embryologic development, tissue growth, tissue repair, and regeneration due to its pluripotency and immunomodulatory capacity (Scuderi et al., 2013; Dai et al., 2016; Tabatabaei Qomi and Sheykhhasan, 2017). However, its role in end-organ homeostasis and repair following organ damage remains largely undefined.

The central aim of regenerative medicine is to restore function to pathological tissues via cellular regeneration and reimplantation. Although recent advances in gene therapy have enabled researchers to modify the human genome for therapeutic purposes, its utility is limited by the irreversible nature gene editing (Bates, 2016). Furthermore, most human diseases cause myriad transcriptional effects, such that targeting a subset of genomic loci is likely insufficient to successfully regenerate function (Hong, 2018). Regenerative medicine thus turns also to the procurement of adult mesenchymal cells, including ASCs, for *in vitro* expansion, differentiation into end-organ tissues, and eventual reimplantation (Shyam et al., 2017).

Among the biologic mechanisms capable of influencing cellular plasticity, epigenomic modification has been shown to impact both the regenerative capacity and eventual differentiation of adult stem cells (Ceccarelli et al., 2018; Costantino et al., 2018). These molecular marks, including methylation of cytosine residues, have been described as critical features in determining the process of ASC aging and senescence (Munoz-Najar and Sedivy, 2011). Identification of epigenetically active small molecules, termed epidrugs, has enable the exogenous manipulation of DNA methylation to define both the transcriptional and phenotypic components under direct epigenetic control, targeting gene expression in a transcriptome-wide manner.

5′-azacitidine (5′-AZA) is one such epigdrug, that disrupts the methylation of cytosine when incorporated into the newly synthesized DNA of progeny cells (Sajadian et al., 2015). Its use *in vitro* has been shown to trans-differentiate non-osteoblastic cells into an osteocytic lineage (Cho et al., 2014). 5′-AZA has also been shown to attenuate aging-associated impairments in proliferation of ASCs (Kornicka et al., 2017). Similarly, reduction of genome-wide DNA methylation has been linked to enhancement in their self-renewal (Kornicka et al., 2017).

In the current study, we investigate how 5′-AZA-induced alterations in genome-wide DNA methylation likely affect molecular networks involved in proliferation and slowing of senescence processes in ASCs. We use reduced-representation bisulfite sequencing (RRBS) to localize differentially methylated regions (DMRs) and identify candidate methylation-sensitive transcriptional regulators of ASC senescence and proliferation.

## Materials and Methods

### Ethics Statement

Human subcutaneous abdominal adipose tissue was collected from abdominal wall resection of two healthy subjects who underwent cosmetic surgery, male and female Caucasian Italians (BMIs < 25) ages 62 and 52 years, respectively. Written informed consent was obtained, and the clinical protocol was approved by the Institutional Review Board of the Department of Experimental Medicine, Sapienza University of Rome (Italy). All human genome-wide DNA methylation data have been uploaded to the NCBI Gene Expression Omnibus database (GSE139157): https://www.ncbi.nlm.nih.gov/geo/query/acc.cgi?acc=GSE139157.

### ASC Isolation and Culture

**FIGURE 1:**
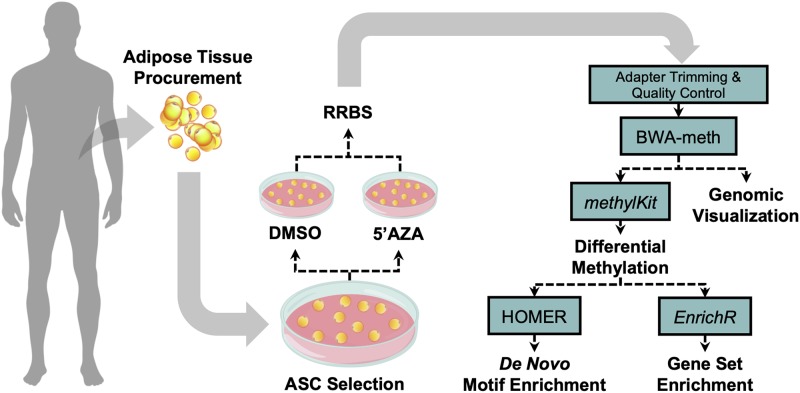
Visual illustration of sample processing and data analysis pipeline. Abdominal white adipose tissue was biopsied from two patients, and subsequently plated for adipose-derived mesenchymal cell selection and treatment with 5′-AZA or vehicle (DMSO) for 24 h. Isolated DNA was bisulfite-reduced and sequenced, followed by bioinformatic analysis of differential CpG methylation.

The tissue procurement, cellular treatment, and data analysis pipeline was performed as illustrated in Figure 1. Adipose tissue was transferred to the laboratory and processed under sterile conditions within 24 h. Isolation of ASCs was performed as previously described (Ceccarelli et al., 2018). Briefly, tissue fragments were washed extensively with sterile phosphate-buffered saline containing 2% penicillin/streptomycin and minced. The extracellular matrix was digested with 0.075% collagenase type I for 30–60 min at 37°C and 5% CO_2_. The suspension was filtered to remove debris and centrifuged for 5 min at 2000 rpm. The pellets of stromal vascular fraction (SVF) containing ASCs were washed with PBS, then resuspended in the culture medium and transferred to a culture flask. ASCs were self-selected out of the SVF, since they were adherent to the plastic tissue cultureware. ASC cells were cultured in DMEM-Ham’s F-12 medium (vol/vol, 1:1) (DMEM/F12; Gibco) supplemented with 10% FBS, 100 U/ml penicillin, 100 mg/ml streptomycin, and 2 mM L-glutamine, and maintained in a 5% CO_2_ incubator at 37°C in a humidified atmosphere, with medium change twice a week. When reaching 80–90% confluence, cells were detached with 0.5 mM EDTA/0.05% trypsin (Gibco) for 5 min at 37°C and then replated. ASCs were expanded and cell viability was assessed by using the trypan blue exclusion assay. Cell morphology was evaluated by phase contrast microscopy. Experiments were conducted between passage numbers 7,8. Absence of mycoplasma contamination was confirmed by PCR with specific primers.

### Treatment of ASC With 5′-Azacytidine (5′-AZA)

The DNA methyltransferase inhibitor 5′-AZA was purchased from Sigma-Aldrich (Milan, Italy) and was reconstituted at 10 mM using dimethyl sulfoxide (DMSO). ASCs were seeded at a cell density of 5 × 10^3^ cells/cm^2^. After cell attachment, the medium was changed to freshly made culture medium containing 10 μM 5′-AZA. After 24 h, the 5′-AZA containing culture medium was refreshed for another 24 h (total treatment 48 h). Control samples were treated with DMSO alone at 0.1% (v/v) concentration.

### DNA Extraction

Pooled genomic DNA was isolated from ASCs treated with or without 24-h of 10 μM 5′-AZA using the GeneJET Genomic DNA Purification Kit (Thermo Scientific). DNA concentration and purity were determined using Qubit 3 Fluorometer (Invitrogen) and NanoDrop 2000 (Thermo Scientific). DNA integrity was checked on 1% agarose gel.

### Reduced-Representation Bisulfite Sequencing

Sequencing was performed at the Genomix4Life S.r.l. with subsequent bioinformatics performed at the University of Alabama at Birmingham (United States). Briefly, 2 μg of genomic DNA were used for each library preparation. Each DNA sample was digested by MspI restriction enzyme. The digested products were purified with the GeneJet PCR Purification Kit (Thermo Fisher Scientific) and libraries were prepared by TruSeq Library Prep Kit (Illumina, Inc., United States). Fragments were bisulfite converted using the EZ DNA Methylation-Gold Kit (Zymo Research, United States). The converted DNA was amplified using PfuTurbo Cx Hotstart DNA Polymerase (Agilent Technologies, United States). The amplified fragments were purified by AMPure XP Beads and further quantified by the Agilent 4200 TapeStation (Agilent Technologies, United States). Each DNA library was analyzed by paired-end sequencing read (2 × 75 cycles) on Illumina Nextseq 500.

### Bioinformatic Analysis and Data Visualization

#### RRBS Analysis

Details of the R coding scripts and other bioinformatics tools used in the current study are available as online Supplemental Methods and GitHub data repository: https://github.com/mepepin/Napoli_ASCs. To evaluate sequencing quality, *FastQC* (0.11.7) was used both before and after adapter trimming via *TrimGalore* (0.4.4). The bisulfite-reduced and sequenced reads were then aligned to the CT- and GA-converted human hg38 (GRCh38.p12) genome assembly via BWA-meth to quantify relative alignment of methylated and unmethylated CpGs, respectively (Sun et al., 2018). We then quantified differential DNA methylation in 500-base windows to exploit the regional CpG methylation analysis afforded by RRBS using the R package *methylKit* (1.8.0). Briefly, alignments were first filtered for those with <99.9% methylated CpGs and sequencing depth >10× to remove PCR-biased and low-coverage CpG sites, respectively. To determine regional methylation, 500-base genomic window was used to perform regional methylation in ASCs treated with 5′-AZA relative to vehicle-treated ASCs. Statistical significance of differential-methylation was assumed based on an over-correction adjusted Fisher’s exact test, as recommended by Wreczycka et al. (2017). A sliding linear model (SLIM) method was used to adjust *P*-values for multiple testing (Wang et al., 2011).

#### Pathway Enrichment Analysis and Functional Network Mapping

Functional gene set enrichment analyses (GSEA) were performed using the interactive web-based platform *Enrichr* (Chen et al., 2013) using the Kyoto Encyclopedia of Genes and Genomes (KEGG) pathway database (Kanehisa, 2002). Weighted gene network analysis and visualization were performed using Cytoscape (3.7.0) using genes with differentially methylated promoters (DMPs) and a low-stringency statistical threshold of *P* < 0.05. Heatmap and hierarchical clustering generation was performed using *pheatmap* package (1.0.8) within R.

### Statistical Analysis

Unless otherwise indicated, statistical significance was determined via unpaired two-tailed Bonferroni-adjusted *P*-value (*Q*) < 0.05. Pathway analysis and motif enrichment both employed an unadjusted *P*-value using the Fisher’s exact test.

## Results

### Differential Methylation of ASCs Treated With 5′-AZA

**FIGURE 2:**
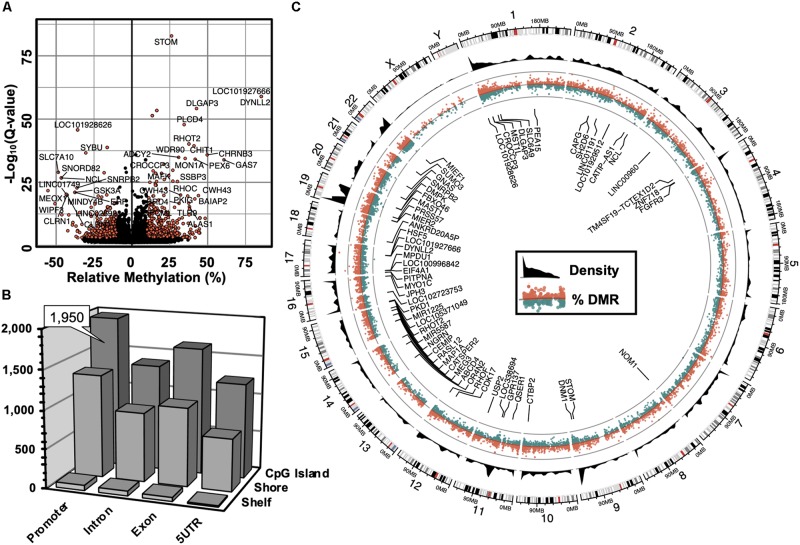
Genome-wide alterations in DNA methylation. **(A)** Volcano plot illustrating -log_10_(*Q*-value) as a function of differentially methylated regions (DMRs, in % difference). Genes were labeled which contained robust changes in methylation (*Q* < 10^– 15^), | Methylation| > 25%). **(B)** 3-dimensional bar plot depicting the distribution of DMRs according to both genic annotation (promoter, intron, exon, and 5′ untranslated regions) and proximity to CpG Islands (CpG Island, Shore, and Shelf). **(C)** Circular plot illustrating the genomic distribution of DMRs (red = hyper-methylated, green = hypo-methylated), and DMR density (black). Labeled genes are DMRs (| Methylation| > 10%) that meet genome-wide significance (Q < 10^– 8^).

The computation of differentially methylated regions (DMRs) identified 4,797 DMRs (*Q*-value < 0.05) associated with 3,625 annotated genes (Supplementary Table S1), among which was an equivalent proportion of hyper-methylated (2,588) and hypo-methylated (2,209) regions was uncovered. A volcano plot was used to examine the genes associated with differentially methylated promoters with 5′-AZA treatment (Figure 2A). Because the transcriptional effects of DNA methylation are highly dependent on the position of DMRs relative to known genomic features, with promoter methylation classically inversely associated with transcriptional activity (Bird, 1986; Jjingo et al., 2012), we examined the distribution of DMRs in relation to genomic locations most impacted by changes in DNA methylation following treatment with 5′-AZA. Annotated DMRs were mapped onto both genic location (promoter, 5′UTR, gene body, and 3′UTR) as well as according to their distance from established CpG Islands (CGIs). Methylation dynamics were modestly concentrated among CpG islands located in the promoter region (Figure 2B). Illustrating DMRs using a circular genome plot demonstrated the genome-wide distribution of methylation changes, numerous hyper-dynamic regions, and a broad array of genes with DMRs reaching genome-wide significance (*P* < 10^–8^) (Figure 2C).

### Pathway Enrichment Analysis of DMRs

**FIGURE 3:**
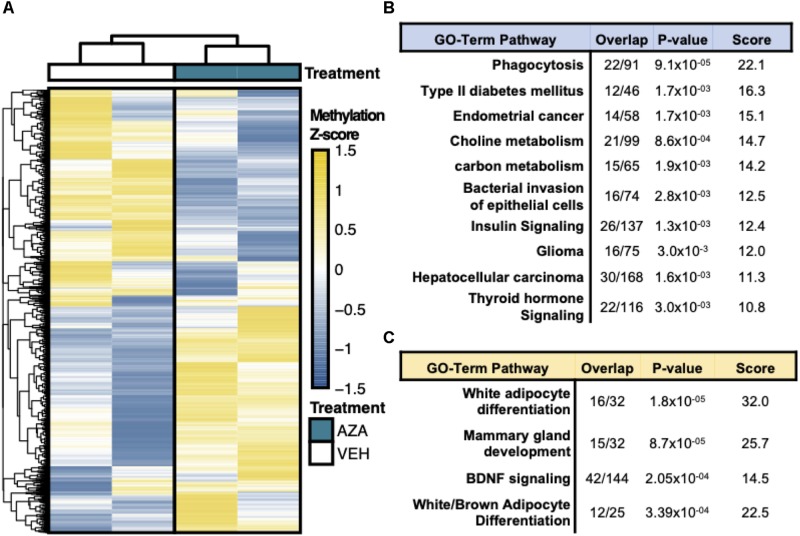
Reduced Representation Bisulfite Sequencing Analysis. **(A)** Hierarchical clustering and heatmap analysis of regional differential CpG methylation (DMRs).* **(B)** GO-term enrichment analysis of hyper-methylated and **(C)** hypo-methylated DMRs demonstrating the % enrichment (“Overlap”), *P*-value of overlap by Fischer exact test, and composite enrichment score.

Hierarchical clustering and heatmap visualization was then used to understand the degree of consistency between 5′-AZA and DMSO-treated ASCs, which revealed robust separation of differential promoter methylation between 5′-AZA and vehicle-treated ASCs with no apparent outliers (Figure 3A). Gene set enrichment analysis (GSEA) was performed using the genes possessing differentially methylated promoters, separating the hyper-methylated from hypo-methylated DMRs to interpret the possible impact of methylation dynamics on the enriched pathway(s). This approach identified phagocytosis (*P* = 10^–5^), type 2 diabetes (*P* = 10^–4^), and numerous metabolic pathways as disproportionately impacted by promoter hypo-methylation (Figure 3B and Supplementary Table S2), whereas white-adipocyte differentiation was the top most-enriched pathway among genes with hyper-methylated promoters (Figure 3C and Supplementary Table S3). Altogether, these findings suggest that 5′-AZA-mediated promoter methylation influences the metabolic phenotype of ASCs.

### Weighted Functional Network Enrichment Analysis

**FIGURE 4:**
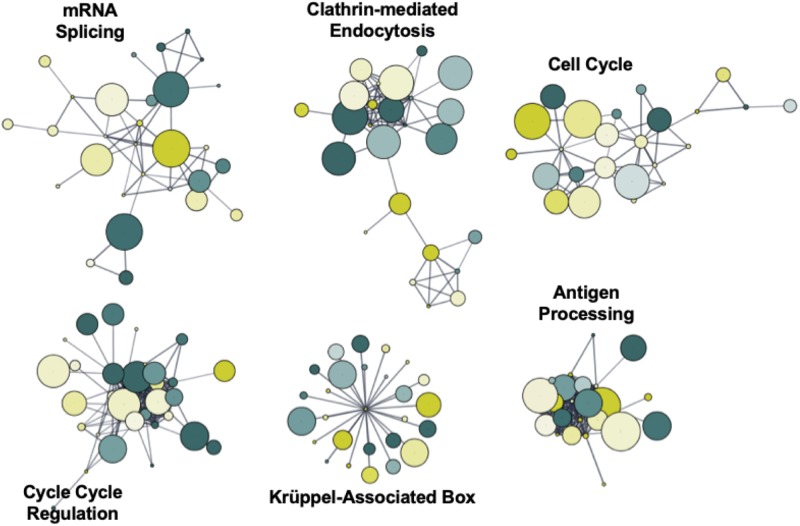
Functional network enrichment analysis. Weighted gene co-expression network analysis of DMRs based on protein-protein interactions found within the STRING database. Network topology was defined according to both the degree of interaction and number of common nodes (minimum of 3) using a Markov cluster algorithm (MCL).

To identify novel networks affected by 5′-AZA treatment, we used the STRING database (Szklarczyk et al., 2017) to generate a network of DMRs based on known and predicted interactions among their encoded proteins, with Markov Clustering (MCL) to separate DMR networks according to both the degree and number of adjacent interactions (Figure 4). After ranking nodes by degree of interaction, the largest cluster was identified to functionally enrich pathways via the KEGG pathway database associated with cellular proliferation and developmental programs: krüppel-associated box (*P* = 2.9 × 10^–32^), antigen processing (P = 6.9 × 10^–26^), mRNA splicing (*P* = 1.1 × 10^–23^), cell cycle regulation (*P* = 5.7 × 10^–17^), clathrin-mediated endocytosis (*P* = 1.2 × 10^–15^), and cell cycle (BH-adjusted *P* = 2.9 × 10^–13^). Because we and others have previously found Krüppel-like factors (KLFs) to possess methylation-sensitive response elements (Pepin et al., 2019), the current analysis further supports that 5′-AZA treatment is sufficient to induce disproportionate differential methylation of KLF target gene promoters.

### Motif Interference by DNA Hyper-Methylation

**FIGURE 5:**
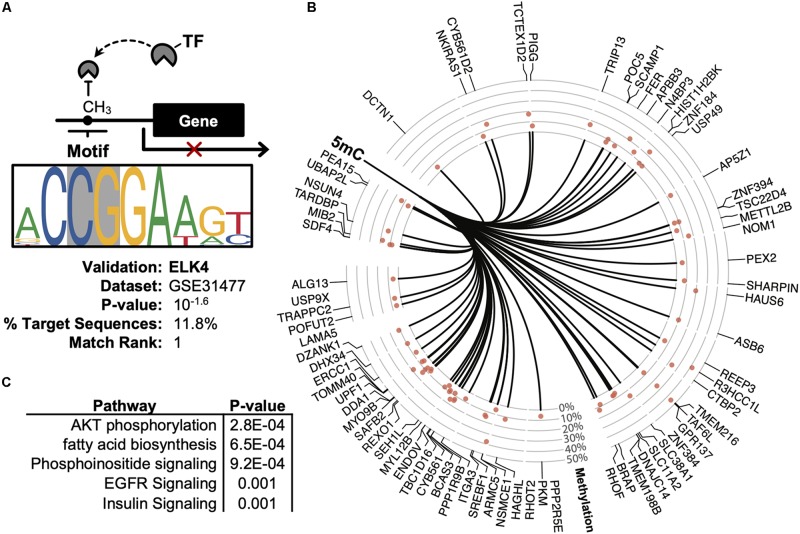
Promoter-based DMR enrichment of known response elements. **(A)** Known motif enrichment of hyper-methylated DMRs localized to the promoter region based on the ENCODE database of ChIP-sequencing datasets. **(B)** Downstream ELK4 targets genes with hyper-methylated (Q < 0.05, | methylation| > 5%) promoters based on the ENCODE ChIP-sequencing dataset by Cayting et al. (GSE31477). **(C)** Gene-set enrichment analysis of ELK4 gene targets with hyper-methylated promoters, showing the top-5 disproportionately enriched pathways.

Promoter DNA methylation regulates gene expression in-part by interfering with the binding of transcription factors, particularly those possessing CpG-rich response elements (Medvedeva et al., 2014). Therefore, to identify candidate transcription factors likely disrupted by co-localizing promoter methylation, a *de novo* motif enrichment was performed via HOMER (Heinz et al., 2010). To correct for the CpG density of DMRs, background genomic regions were selected according to CpG content when performing hypergeometric enrichment. From this analysis, we found that *ZNF711* was significantly associated with hypo-methylated promoter DMRs (Log_10_(*P*) = −9.9) (Supplementary Figure S1A), whereas the ELK4 motif disproportionately co-localized among hyper-methylated promoters (Figure 5A). The ENCODE dataset (GSE31477) was then used to identify genes associated with hyper-methylated DMRs (*Q* < 0.05, |Methylation| >10%) found in the promoter of ELK4 target genes, revealing (Figure 5B). Gene-set enrichment of co-localized DMRs within ELK4 target promoters identified AKT signaling as disproportinately affected by DMR co-localization, supporting prior studies demonstrating the role of ELK4 and HDAC (Figure 5C). A preliminary evaluation of ELK4 expression revealed its induction in ASC’s treated with 5′AZA (Supplementary Figure S1B). Taken together, these observations support the hypothesis that 5′-AZA-induced promoter methylation may interfere with the downstream targets of ELK4 signaling and may therefore disrupt its role as a regulator of cellular proliferation (Boog, 1993).

## Discussion

The epigenetic programs that define cellular identity offer fundamental insight into the mechanisms of cellular regeneration. These developmental programs, though once considered irreversible, have become a major focus of investigation as researchers seek to understand the pathogenesis of disease, tissue regeneration, and cellular aging. The field of regenerative medicine has begun to embrace a new treatment paradigm wherein senescent end-organ cells may be harvested from patients and re-programmed for therapeutic purposes. However, progress in regenerative medicine is currently limited by an incomplete understanding of the cellular machinery and molecular programs responsible for determining cell fate. In the current study, we employed RRBS to uncover an epigenomic program within ASC’s which encodes proliferation and senescence in response to 5′-AZA.

Motif analysis of hyper-methylated DMRs uncovered the disproportionate methylation of promoter targets for ETS domain-containing transcription factor ELK4. As a regulator of numerous proto-oncogenes, ELK4 is believed to regulate malignant transformation in the context of multiple cancer types (Wu et al., 2018). Specifically, ELK4 has been shown to recruit and stabilize epigenetic regulator SIRT7 to promote tumor growth and maintain rapid cellular proliferation (Makkonen et al., 2008; Day et al., 2011). Therefore, we provide novel support that differential DNA methylation of the ELK4 response element modulates the downstream effects of ELK4 signaling.

Overall the current study has identified 4,797 genomic regions differentially methylated in 5′-AZA-treated relative to vehicle-treated ASCs. Furthermore, differential methylation was found to disproportionately affect promoter-associated CpG Islands, regions which have been extensively studied for their role as negative transcriptional regulators (Bird, 1986; Jjingo et al., 2012). Roughly half of these DMRs were hyper-methylated, and therefore cannot be explained by the direct effects of 5′-AZA, a potent inducer of DNA demethylation (Christman, 2002). Numerous potential mechanisms exist capable of mediating these indirect effects, including the compensatory regulation of DNA methyltransferase (DNMT) activity. Furthermore, 5′-AZA has been found to regulate DNA methylation in a targeted manner (Tabolacci et al., 2016). Such genomic restriction could involve the pre-templated epigenetic architecture present prior to 5′-AZA treatment; however, such notions remain speculative, warranting future studies to empirically define.

Although we provide numerous novel findings, we must acknowledge the key limitations of the current analysis. Although genome-wide approaches represent a valuable tool to identify novel candidates and genomic networks, our pooled analysis of ASCs yields a small sample size that limits the statistical generalizability of our DNA methylation analysis. Future work will involve single-cell analytical approaches to appreciate spectrum of response to 5′-AZA treatment. Additionally, future work is needed to correlate epigenomic changes with a cellular phenotype. Furthermore, we cannot exclude that other epigenetic phenomena influence adipocyte aging and proliferation. Lastly, validation and functional studies using larger patient cohorts are needed to assess the *in vivo* efficacy of our epigenetically modified ASCs in preclinical models to assess their usefulness in regenerative medicine.

## Conclusion

In the current study, we offer several important, albeit preliminary, insights that support the existence of a complex milieu of epigenomic changes able to regulate proliferative gene programs. Although future studies are needed to understand the therapeutic potential of these novel programs in ASCs, we show that 5′-AZA activates a methylation program that likely interferes with *ELK4* downstream signaling, thereby offering one potential mechanism whereby DNA methylation influences transcriptional activity toward adipocyte differentiation pathways.

## Data Availability Statement

All data generated or analyzed during this study are included in this article and are found on the NCBI Gene Expression Omnibus (GEO) repository (GSE139157), as well as at the following repository: https://github.com/mepepin/Epigenetics-in-ASCs.

## Ethics Statement

Written informed consent was obtained, and the clinical protocol was approved by the Institutional Review Board of the Department of Experimental Medicine, Sapienza University of Rome (Italy).

## Author Contributions

MP designed the bioinformatics analysis, interpreted the data, drafted the manuscript, and designed illustrations used in the current work. TI, GB, and CS interpreted the data and drafted the manuscript. MM and EA provided technical experimental support and interpreted the data. SC and FM participated in the design of the study, performed ASC isolation and treatment, and interpreted the data. GD, GF, MF, LD, and GN revised the manuscript critically for important intellectual content. AW provided critical feedback on methods and interpretation, as well as revision of the text. CM and CN designed and coordinated the study and revised the manuscript.

## Conflict of Interest

The authors declare that the research was conducted in the absence of any commercial or financial relationships that could be construed as a potential conflict of interest.

## Abbreviations

5 ′ -AZA: 5 ′ -azacitidine
AKT: protein kinase B
ASCs: Adipose-derived mesenchimal stem cells
DMR: differentially methylated 500-base CpG-containing region
ELK4: ETS transcription factor.
