# Supplementary material for: Differential DNA Methylation Encodes Proliferation and Senescence Programs in Human Adipose-Derived Mesenchymal Stem Cells
Source: Front Genet. 2020 Apr 15;11:346. doi: 10.3389/fgene.2020.00346 (PMC7174643; doi:10.3389/fgene.2020.00346)
Supplement: Supplementary file 1 [file Image_1.pdf]

Figure S1

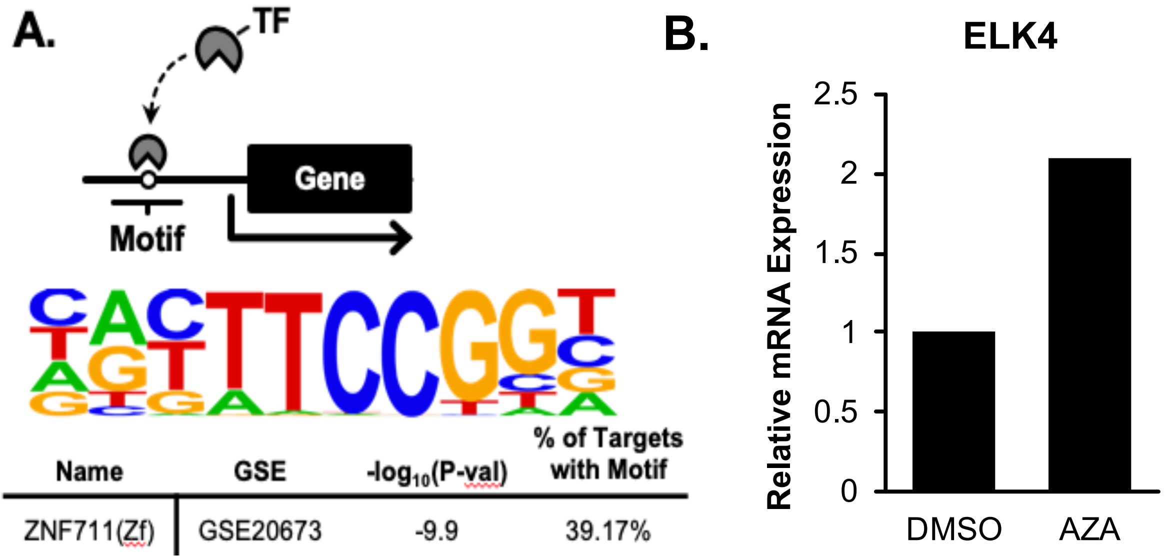

Figure S1: motif enrichment analysis. A. known motif enrichment using hypo-methylated promoter DMRs ($P < 0.05$) identifying ZNF711. **B.** ASCs were isolated from 2 additional subjects (females, BMI < 25, ages 63 and 42), and real-time PCR quantification of ELK4 gene expression in ASC's treated with 5'AZA relative to DMSO for 48 hrs (pooled n = 2).

Fig. 1B Methods: ASCs isolated from adipose tissue collected from two female donors (BMI < 25, ages 63 and 42), were cultured as indicated above, and at passage numbers 5 - 7 were treated with 10 μM 5-aza for 48 hrs. Control samples were treated with DMSO alone at 0.1% (v/v) concentration. Total RNA from cell cultures was extracted using TRIzol reagent (Invitrogen by Life Technologies, Carlsbad, CA, USA) following the manufacturer's instructions. RNA samples were quantified using a NanoDrop spectrophotometer (ThermoFisher Scientific) cDNA was generated with oligo (dT) from 1 μg of RNA using the SuperScript III Reverse Transcriptase Kit (Invitrogen). Pooled cDNAs from the two patients were used for amplification of ELK4, using the appropriate TaqMan gene expression assay probes (Applied Biosystems by Life Technologies). The assays were conducted in triplicate on an ABI 7500 real- time instrument (Applied Biosystems) using the following conditions: 95° C for 10 min, then 95° C for 15 sec and 60° C for 1 min, repeated 40 times. Relative quantification was performed using Cyclophilin A (PPIA) mRNA as an endogenous control, as previously reported (Ceccarelli et al., Stem Cells Int, 2018). Data were analyzed with PRISM7.
